# Association between Periodontitis and Pulp Calcifications: Radiological Study

**DOI:** 10.1155/2022/9599554

**Published:** 2022-08-22

**Authors:** Bassim Nissrin, Rezki Basma, Sakout Majid

**Affiliations:** ^1^Department of Conservative Odontology, Faculty of Dentistry, Mohamed V University, Ibn Sina Center for Dental Consultation and Treatment (CCTD), Rabat, Morocco; ^2^Private Practice, Rabat, Morocco; ^3^Department of Conservative Odontology, Faculty of Dentistry, Mohamed V University, Mohamed V Military Teaching Hospital, Rabat, Morocco

## Abstract

**Objective:**

The presence of intrapulpal calcifications is one of the effects reported as a consequence of periodontal pathology. Although the impact of the pulp pathology on the periodontium is obvious, the contrary remains unclear and controversial. This study was conducted in order to better understand this fact and establish a potential association between periodontitis and intrapulpal calcifications and then to determine the factors associated with their occurrence.

**Materials and Methods:**

To investigate the issue, a retrospective radiological study using periapical preoperative radiographics assessed 332 teeth taken from the records of 79 patients who received treatment for periodontitis. In the second part of the study, 81 of the sample with intact dental crowns presenting an attachment loss were compared to their contralateral with intact dental crowns without any attachment loss. The study of the association between periodontitis and intrapulpal calcifications and the factors associated with their occurrence was performed by the Chi squared and Fisher's exact tests. The significance level was set at 0.05.

**Results:**

The results indicated that 251 (75.6%) teeth had an attachment loss while 102 (30.7%) had intrapulpal calcification. Among the 206 (62%) teeth with intact crown, only 6 (1.8%) showed calcification in the pulp cavity and 20 (6%) showed calcification in the root canals, with a statistically significant difference (*p* < 0.005) compared to teeth with restorations and caries. For the 32 (19.7%) teeth with coronary calcification, 18 (22.2%) presented an attachment loss versus 14 (17.2%) without attachment loss; the difference was not statistically significant (*p*=0.6). Similarly, only 13 (16%) of a total of 22 (13.5%) teeth with root canal calcification had attachment loss versus 9 (11.1%) without attachment loss. This difference was not statistically significant (*p*=0.5).

**Conclusion:**

This radiographic study revealed no association between the presence of periodontitis and the occurrence of intrapulpal calcifications. Although intrapulpal calcifications were present in some teeth with loss of attachment, they were not necessarily the consequence of periodontal disease.

## 1. Introduction

The endodontium and the periodontium represent two closely related entities that are well illustrated by the expression “endo-periodontal continuum.” There is a close relationship between the pulp and the periodontium. These two structures can communicate through physiological (apical, lateral, and accessory canals, tubuli, etc.) or pathological (fractures, cracks, etc.) ways. This not only allows to maintain vascular, nervous, and sensorial relationships but also promotes the reciprocal transmission of inflammatory and degenerative phenomena, which explains the pulpo-peridontal pathological interactions [2–4].

Although the impact of pulp pathology on the periodontium is very evident, the contrary remains unclear and very controversial [5]. Several histological [6–10] and radiographic [11–14] studies have attempted to identify histological changes in the pulp when periodontal disease is present and the results were of great variety, varying from normal pulp to total necrosis including chronic inflammation and degenerative manifestations.

These degenerative manifestations can appear as pulp calcifications, defined as intrapulpal mineralized deposits under the form of nodular calcifications generally found in the pulp cavity or diffuse calcifications found in the root canals [4, 15, 16].

In the literature, several other factors were associated with the occurrence of intrapulpal calcifications, such as the state of the dental crowns [13, 16–18], age [12, 19, 20], and sex [11, 13, 14]. In fact, when carious lesions or restorations are present, the pulp responds protectively to this chronic irritation by forming secondary dentin, resulting in a decrease in the size of pulp chamber and degenerative diffuse calcification. Moreover, pulp senescence is accompanied in the elderly by the formation of secondary and tertiary dentin, by a decrease in pulp cells and an increase in fibrosis, which may be associated with the presence of pulp calcification.

The high diversity of reported results increases the need for more investigations to achieve clinically useful results and conclusions. For this purpose, this study was conducted to identify a possible association between periodontitis and intrapulpal calcifications and then to determine the factors associated with their occurrence.

## 2. Materials and Methods

The study was conducted in compliance with the ethical principles stated in the Declaration of Helsinki and was approved by the Ethics Committee for Biomedical Research, Mohammed V University in Morocco under ID no. 41/22.

This retrospective radiographic study was carried out between December 2016 and February 2017 at the Ibn Sina Center for Dental Consultation and Treatment (CCTD), Faculty of Dentistry, Mohammed V University in Rabat, Morocco.

### 2.1. The Study Sample

The study sample size was calculated using the following formula: *n* = *Z*^2^*P*(1 − *P*)/*D*^2^.

The prevalence by subjects (*P*) of pulpal calcification using periapical radiographs was estimated at 12% [14]. The *Z*-value was 1.96 for 95% confidence interval, and the precision level *D* was determined at 0.05. From the above formula, the minimum size required was 162 patients.

Of the 165 periodontitis patient records analyzed, only 79 records meeting the inclusion criteria were included in the study. From these records, 332 teeth (single and multiple root teeth) were evaluated.

### 2.2. Inclusion Criteria

The study included records of periodontitis patients of both sex over 17 years old. A patient with at least two nonadjacent teeth with a clinical interdental attachment loss (CAL) greater than 1 mm was considered as a periodontitis patient. This attachment loss was assessed from the cementoenamel junction to the bottom of the periodontal pocket.

All preoperative periapical radiographs of teeth with or without periodontal disease, intact, or with carious lesions or restorations with various pulp conditions were examined.

### 2.3. Exclusion Criteria

Records with incomplete clinical information, poor-quality overlapping radiographs, or radiographs with crowns or bridges that did not allow a clear view of the pulp cavity, and teeth that had undergone endodontic treatment were excluded from the study.

### 2.4. Data Collection

From the patient's records, the following clinical data were collected and adapted according to the new classification of periodontal diseases [21]:

- Sex and age
- Medical state

#### 2.4.1. Stages of the Periodontitis

- Stage I: an initial periodontitis where CAL at the site of greatest loss was 1 to 2 mm.
- Stage II: a moderate periodontitis where CAL at the site of greatest loss was 3 to 4 mm.
- Stages III and IV: a severe periodontitis with CAL at the site of greatest loss was ≥5 mm.

#### 2.4.2. Grades of Periodontitis

- Grade A (slow progression) and grade B (moderate progression) which corresponded to the chronic periodontitis in the previous classification of periodontitis [22], where there is a low loss attachment and periodontal destruction commensurate with biofilm deposits.
- Grade C (rapid progression) which corresponds to aggressive periodontitis in the previous classification. In this category, the CAL and periodontal destruction exceed expectation, given biofilm deposits; there are specific clinical features evocative of periods of rapid progression and/or early onset disease (e.g., molar/incisor pattern) and it is related to risk factors, such as diabetes and smoking.

#### 2.4.3. State of the Dental Crowns of the Assessed Teeth

The state of assessed teeth varied from intact to decayed, with adequate or inadequate restoration.

The radiographs studied included analogical periapical films (Kodak, Eastman Kodak company, NJ, USA) taken using the paralleling technique along with film holders (Rinn XCP, Dentsply Sirona, NC, USA). These radiographs were examined on a dental X-ray viewer (Titanox, Limassol, Cyprus) using a magnifying lens by one examiner in the Department of Endodontics. Uncertain situations were discussed with two other observers. The tooth was considered to have calcification in the pulp cavity if it had nodular calcification, pulp retraction, or a ceiling close to the pulp bottom.

The tooth was considered as presenting calcification at the root canals if these were constricted or have shown a disappearance of the root canal lumen at the apical, middle, or coronary third.

Thirty radiographs were evaluated again by the same examinator one week later. The intraexaminer's reliability was assessed according to Cohen's kappa coefficient and was calculated as 0.96.

**Figure 1:**
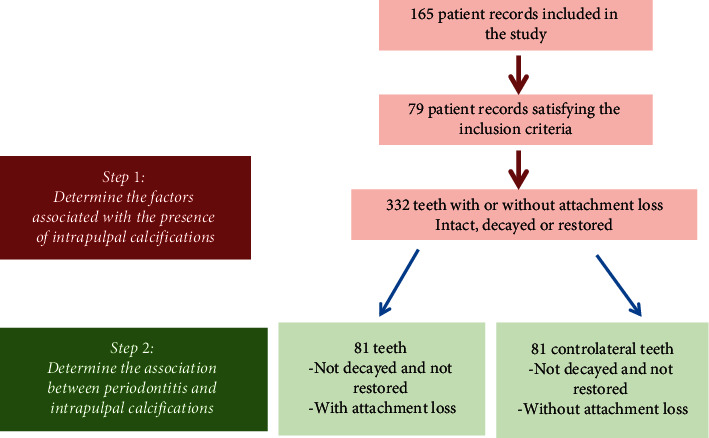
Flowchart of the study.

In the second part of the study, 81 existing teeth with intact crowns (without carious lesions or restorations) with attachment loss were compared with their contralateral without attachment loss (Figure 1), in order to evaluate the association between attachment loss and pulp calcification by reducing confounding factors, such as crown state, sex, and age.

### 2.5. Statistical Analysis

The results were analyzed using the software (SPSS 13.0, Inc., Chicago, IL, USA). The qualitative variables were expressed in terms of number and percentage. The study of factors associated with the presence of intrapulpal calcifications was assessed by the Chi squared test and the exact Fisher's test. The study of the association of the attachment loss with the presence of intrapulpal calcifications was performed by the Chi squared test. The significance level was fixed at 0.05.

## 3. Results

The analysis of patient records (Table 1) shows that 47 (59.9%) patients were female, only 8 (10.3%) patients were over 50 years, and only 13 (16.9%) patients had grade C periodontitis.

Table 2 shows the dental and periodontal characteristics of the teeth examined in the study. Two hundred fifty-one teeth (75.6%) had an attachment loss, 102 (30.7%) had intrapulpal calcification either at the pulp cavity 81 (24.4%) or at the root canal 61 (18.4%).

Table 3 analyses the factors associated with the presence of intrapulpal calcifications in the pulp cavity and root canals. Among the 206 (62%) intact teeth (without carious lesions and restorations), only 6 (1.8%) teeth showed calcification in the pulp cavity and 20 (6%) teeth showed a calcified deposit in the root canal, with a statistically significant difference (*p* < 0.005).

Table 4 analyses the association between the attachment loss and the presence of intrapulpal calcifications. Among the 32 (19.7%) teeth with coronary calcification, 18 (22.2%) teeth presented an attachment loss versus 14 (17.2%) without attachment loss, and this difference was not statistically significant (*p*=0.6).

Similarly, among the 22 (13.5%) teeth with intracanal calcification, 13 (16%) had an attachment loss versus 9 (11.1%) teeth without attachment loss, and this difference was not statistically significant (*p*=0.5).

## 4. Discussion

### 4.1. Association between Attachment Loss and Pulpal Calcification

By comparing the calcification rate on teeth with and without an attachment loss, no statistically significant difference between the two groups was found. Among the 81 (24.4%) teeth with intrapulpal calcification in the pulp cavity, only 65 (19.5%) had an attachment loss and among the 61 (18.3%) teeth with calcific root deposition, only 51 (15.3%) had an attachment loss. This result is in concordance with that of Ghoddusi et al. (2003)[8] and also Fatemi et al. (2012) [23], who reported that there is no association between the presence of an attachment loss and the presence of pulp calcifications.

Other studies [7, 24–26] have also reported the absence of the influence of periodontal disease on the pulp state. This was explained by Stallard et al. (1968) [27], who considered that the cementum would prevent the passage of septic contents from the periodontal pocket to the pulp. And even if the progressive destruction of the periodontal attachment system brings the root surfaces and the septic contents of the periodontal pockets into direct contact, the microorganisms of the dental plaque, the bacterial toxins, and the substances resulting from the inflammation of the periodontium will not be able to pass through the cementum barrier into the endodontium but will be able to evacuate easily through the coronal part of the pocket.

In contrast to the findings of this study, other authors have established an association between attachment loss and the presence of pulp calcifications [19, 28–30]. This result was explained by the fact that periodontal diseases would interfere with the supply of blood and necessary pulp nutrients, leading to a reduction in cellular elements and consequently an increase in calcifications [31]. Other studies have identified the presence of pulp calcifications [32] or a reduction in canal lumen at teeth [33] only at the root pulp of teeth with attachment loss compared to teeth without attachment loss.

Regarding the influence of the grade of periodontal disease on the occurrence of pulp calcifications, among the 81 (24.4%) teeth with pulp calcification in the pulp cavity, 68 (20.8%) were from patients with grade A or B periodontitis, while only 13 (3.9%) were in patients with grade C periodontitis, but this difference was not statistically significant (*p*=0.8)

Similarly, among the 61 (18.4%) teeth with root calcification, 55 (16.5%) of them were from patients with grade A or B periodontitis versus only 6 (1.8%) from patients with grade C periodontitis, but this difference was not statistically significant (*p*=0.09)

This high percentage of intrapulpal calcifications associated with the presence of grade A or B periodontitis could be due to the fact that the majority of the patients included in this study (83.1%) had grade A and B periodontitis.

Concerning the stage (severity) of the periodontitis, no statistically significant association was found (*p*=0.1) between the stage of periodontal disease and the presence of intrapulpal calcifications, either in the pulp cavity or in the root canals.

This finding has also been reported by other authors [10, 15, 24, 25], who found no significant difference in the number of pulp calcifications in moderate (stage II) versus severe (stages III and IV) periodontitis.

Some studies [7, 18] have reported that there are pulps with normal histological aspects in periodontitis of varying severity. The pulp condition could be completely independent of the type and severity of the periodontitis, and the degree of pulp disease does not necessarily reflect the severity of the periodontal disease. Indeed, on many teeth with completely exposed roots, the pulp can be healthy-looking [34].

However, some histological studies [32, 35, 36] have reported an increased incidence of pulp calcifications in the teeth with severe periodontitis, particularly when bone lysis has progressed to the apex.

Other authors reported that pulpal changes increased with the increase in periodontal pockets. The fibrosis was more present in the superficial pockets because the pulp tends to increase collagen production, whereas when the depth of pockets increased, the pulp would react by producing dystrophic calcifications [37].

Furthermore, for others [3, 38], total pulp disintegration can only be observed when the progression of the periodontitis is such that it extends to the apical foramen and the pathological process is terminal.

In the second part of this study, in order to identify the association between the attachment loss and the pulp calcifications without the confounding factors, such as the presence of carious lesions and restorations at the tooth, the age, and the sex; 81 teeth with intact crowns with an attachment loss were compared with their contralateral teeth with intact crowns as well, without attachment loss.

The results showed no statistically significant difference between the two groups of teeth; therefore, no association between the presence of attachment loss and the presence of pulp calcification was found.

This finding is in agreement with the results reported by Sabeti et al. [39] in their histological study comparing 35 intact teeth extracted with severe bone lysis to teeth extracted for orthodontic reasons. In their study, they concluded that severe periodontitis did not significantly affect pulp vitality and pulp calcification.

In contrast to the results of this study, other authors comparing teeth with various severities of attachment loss without caries and restorations [9, 34, 38, 40] reported that periodontal disease can accelerate the pulp senescence process due to its interference with the nutritional supply of the pulp and concluded that periodontitis can cause the development of pulp calcifications and fibrosis [37].

### 4.2. Crown State

The comparison between the groups of teeth with intact crowns and those with carious lesions or restorations have shown that the highest percentage of teeth without intrapulpal calcifications, both in the pulp cavity 200 (60.2%) and in the root canal 182 (54.8%), belonged to the healthy teeth without carious lesions or restorations, and this difference is statistically significant (*p* < 0.001).

This could be explained by the existence of an association between pulp calcification and the presence of carious lesions and restorations and not necessarily the presence of attachment loss. This finding is in agreement with other studies [13, 16–18, 41] reporting a high incidence of calcified masses in pulps with carious lesions or in restored teeth. This may be due to chronic pulpal irritation in both carious and restored teeth. Caries and microleakage around restorations may trigger a defense reaction in the pulpo-dentinal complex, causing pulpal calcifications.

The mechanism of pulp calcification may be similar to the formation of tertiary dentin near irritated odontoblasts. In a previous study by Sener et al. [16], similar results were noted where pulp calcifications occurred in response to long-standing irritants, and the prevalence of pulp stones was higher in decayed or restored teeth and in teeth with both caries and restorations.

However, other studies [14, 42–44] have reported that there is no association between crown state and the presence of pulpal calcifications. It is unlikely that pulp pathology is the only etiological factor for pulp stone formation, because even in very young teeth and developing tooth germs, the presence of pulp stones was reported [45]. A recent theory also includes calcifying nanoparticles in the air as an etiologic factor for pulp stones [46].

### 4.3. Sex

Although, in this study, women had more cameral and pulpal root calcifications than men, this did not appear to be a significant difference (*P*=0.6 and *p*=0.08). That could be explained by the sex factor. A similar result was found in other studies [11, 13, 14, 16, 17, 20, 28, 42, 44, 47–49].

Some authors tend to explain this difference by the fact that bruxism, which is a long-term irritation, is more prevalent among women. Nevertheless, further studies should be undertaken in the future to support or reject this relationship.

### 4.4. Age

Through this study, no significant difference between the different age groups studied was found (*p*=0.6 and *p*=0.7), this could be due to the fact that the majority of the patients studied (≈90%) were younger than 50 years. This is consistent with the results of other authors [11, 13, 16, 35, 47, 48, 50], who observed a uniform distribution of pulp calcifications across age categories. Rubach and Mitchell [15] suggested that age should be considered as an aggravating rather than an initiating factor.

However, other studies [12, 19, 20, 51–53] have noted an increased incidence of intrapulpal calcifications in elderely patients. This could be explained by the addition of secondary and tertiary dentin, by the progressive decrease in the number of pulp cells, and an increase in mucopolysaccharides and fibrous elements that can lead to calcifications.

In contrast to these studies, some others [42] have noted a rate of 19.2% of pulp calcifications in 500 patients aged 12–13 years. Also, in another study of 42 temporary molars, the authors found that the majority of these teeth (95%) had pulp calcifications, demonstrating the lack of correlation between age and the occurrence of these calcifications [18].

Consequently, the only way to determine the effect of age on the development of pulp calcifications is to conduct long-term longitudinal studies based on annual radiographic follow-up of patients. For the time being, it would seem more logical to associate the causality of pulp calcifications with chronic irritation of the dental organ [16].

### 4.5. Calcification Frequency

In this study, 102 (30.7%) of the teeth had cameral and/or root canal pulp calcifications. The literature reports (Table 5) that the frequency of pulp calcifications is highly variable and can reach up to 90%, depending on the type of study and the radiographic incidence used. The histological approach, compared to the radiological method, seems to give more accurate results. This difference was probably due to the fact that pulp calcifications of less than 200 *μ*m in diameter are not visible on radiographs. However, radiological studies remain the only noninvasive detecting method of pulp calcifications [11, 54].

### 4.6. Limitations of the Study

The study has several limitations. It is a retrospective cross-sectional study where the results must be interpreted with caution and the cause-effect relationship between periodontal disease and intrapulpal calcifications cannot be established. In addition, in radiological studies, such as this one, intrapulpal calcifications are not visible if they are smaller than 200 *μ*m. Moreover, detailed pulpal calcification patterns were not noted, and this analysis did not focus on tooth type.

## 5. Conclusion

This radiographic study revealed that there was no association between the presence of attachment loss and the occurrence of intrapulpal calcifications. Intrapulpal calcifications, although present in some teeth with attachment loss, were not necessarily the consequence of periodontal disease. On the other hand, there was a statistically significant association between the crown state and the presence of pulp calcifications independently of the loss attachment.

## Figures and Tables

**Table 1:** Characteristics of the study population (79 patient records).

*n* = 79	Number (%)
Sex	
(i) Male	32 (40.1%)
(ii) Female	47 (59.9%)

Age	
(i) 17–30 years	29 (35.6%)
(ii) 31–40 years	19 (24.4%)
(iii) 41–50 years	23 (29.5%)
(iv) >50 years	8 (10.3%)
Presence of medical pathology	6 (8.1%)
Drug intake	4 (5.1%)

Grade of periodontal disease	
(i) Grade C periodontitis	13 (16.9%)
(ii) Grade A and B periodontitis	66 (83.1%)

Stage of periodontal disease (severity)	
(i) Stage I	21 (27.7%)
(ii) Stage II	42 (54.2%)
(iii) Stages III and IV	16 (18.1%)

**Table 2:** Dental and periodontal characteristics of the examined teeth (*n* = 332).

*n* = 332	Number (%)
Attachment loss	
(i) Absent	81 (24.4%)
(ii) Present	251 (75.6%)

Crown state	
(i) Intact	206 (62%)
(ii) Decayed	84 (25.3%)
(iii) Adequate restoration	20 (6%)
(iv) Inadequate restoration	22 (6.6%)

Presence of tooth calcification	
(i) No	230 (69.3%)
(ii) Yes	102 (30.7%)

State of the pulp cavity	
(i) Normal	251 (75.6%)
(ii) With calcific deposits	81 (24.4%)

State of root canals	
(i) Radiolucent in appearance	266 (80.1%)
(ii) With calcific deposits	61 (18.4%)
(iii) With internal resorption	5 (1.5%)

State of the deep and lateroradicular periodontium	
(i) Normal radiological aspect	313 (94.3%)
(ii) With a periapical radiolucent lesion	17 (5.1%)
(iii) With a lateroradicular radiolucent lesion	2 (0.6%)

**Table 3:** Factors associated with the presence of intrapulpal calcifications.

	Pulp cavity state				Root canal state				
	Normal	With calcific deposits	Total	*P*	Normal	With calcific deposits	Resorption	Total	*P*
Sex									
(i) Female	152 (45.7%)	47 (14.1%)	199 (59.9%)	0.6	162 (48.7%)	32 (9.6%)	5 (1.5%)	199 (60%)	0.08
(ii) Male	99 (29.8%)	34 (10.2%)	133 (40%)		104 (31.3%)	29 (8.7%)	0 (0%)	133 (40%)	

Age									
(i) 7–30 years	91 (27.4%)	32 (9.6%)	123 (37%)	0.6	101 (30.4%)	20 (6%)	2 (0.6%)	123 (37%)	0.7
(ii) 31–40 years	71 (21. 3%)	20 (6%)	91 (27.4%)		74 (22.2%)	17 (5.1%)	0 (0%)	91 (27.4%)	
(iii) 41–50 years	77 (23.1%)	23 (0.9%)	100 (30.1%)		77 (23.4%)	20 (6%)	3 (0.9%)	100 (30.1%)	
(iv) >50 years	12 (3.6%)	6 (1.8%)	18 (5.4%)		14 (4.2%)	4 (1.2%)	0 (0%)	18 (5.4%)	

Attachment loss									
(i) No	65 (19.5%)	16 (4.8%)	81 (24.4%)	0.06	71 (21.3%)	10 (3%)	0 (0%)	81 (24.4%)	0.07
(ii) Yes	186 (56%)	65 (19.5%)	251 (75.6%)		195 (58.7%)	51 (15.3%)	5 (1.5%)	251 (75.6%)	

Grade of PD									
(i) Grade C	43 (12.9%)	13 (3.9%)	56 (16.8%)	0.8	48 (14.4%)	6 (1.8%)	2 (0.6%)	56 (16.8%)	0.09
(ii) Grades A/B	208 (62.6%)	68 (20.8%)	276 (83.1%)		28 (65.6%)	55 (16.5%)	3 (0.9%)	276 (83.1%)	

Stage of PD									
(i) Stage I	63 (18.9%)	29 (8.7%)	92 (27.7%)	0.1	67 (20.1%)	24 (7.2%)	1 (0.3%)	92 (27.7%)	0.1
(ii) Stage II	138 (41.5%)	42 (12.6%)	180 (54.2%)		151 (45.4%)	27 (8.1%)	2 (0.6%)	180 (54.2%)	
(iii) Stages III/IV	50 (15%)	10 (3%)	60 (18%)		48 (14.4%)	10 (3%)	2 (0.6%)	60 (18%)	

Crown state									
(i) Intact	200 (60.2%)	6 (1.8%)	206 (62%)	^*∗∗*^	182 (54.8%)	20 (6%)	4 (1.2%)	206 (62%)	^*∗∗*^
(ii) Decayed	41 (12. 3%)	43 (12.9%)	84 (25.3%)		59 (17.7%)	24 (7.2%)	1 (0.3%)	84 (25.3%)	
(iii) AR	6 (1.8%)	14 (4.2%)	20 (6%)		15 (4.5%)	5 (1.5%)	0 (0%)	20 (6%)	
(iv) IAR	4 (1.2%)	18 (5.4%)	22 (6.6%)		10 (3%)	12 (3.6%)	0 (0%)	22 (66%)	

PD: periodontal disease, AR: adequate restoration, IAR: inadequate restoration, and ^*∗∗*^significant value.

**Table 4:** Association between the presence of intrapulpal calcifications and attachment loss.

Tooth	In pulpal cavity				In root canals			
	Absence of calcification	Presence of calcification	Total		Absence of calcification	Presence of calcification	Total	
With attachment loss	63 (77.7%)	18 (22.2%)	81 (100%)	*P*=0.6	68 (83.9%)	13 (16%)	81 (100%)	*P*=0.5
Collateral tooth without loss attachment	67 (82.7%)	14 (17.2%)	81 (100%)		72 (88.8%)	9 (11.1%)	81 (100%)	
Total	130 (80.2%)	32 (19.7%)	162 (100%)		140 (86.4%)	22 (13.5%)	162 (100%)	

**Table 5:** Some previous studies on the prevalence (or incidence) of pulpal calcification.

Methodology	Year	Investigators	Number of teeth	Prevalence of pulp calcification (%)
Histological studies	1933	Stafne and Szabo [49]	200	46
	1968	Sayegh and Reed [51]	591	32
	1968	Sundell et al [41]	470	11–18
	1993	Arys et al. [18]	42	78
	1997	Hillmann and Geurtsen [52]	332	3–19

Radiological studies	1967	Holcomb and Gregory [54]	881	4
	1982	Tamse et al. [44]	1380	8–57
	1988	Baghdady et al. [42]	6228	12
	1998	Hamasha and Darwazeh [47]	814	22
	1999	Abdallaoui et al. [9]	2340	10
	2002	Ranjitkar et al. [17]	2396	10
	2009	Gulsahi et al. [14]	13474	5

## Data Availability

The data sets generated and/or analyzed during the current study are available from the corresponding author upon reasonable request.
